# Rhizosphere and litter feedbacks to range‐expanding plant species and related natives

**DOI:** 10.1111/1365-2745.13299

**Published:** 2019-10-16

**Authors:** Marta Manrubia, Wim H. van der Putten, Carolin Weser, Ciska (G. F.) Veen

**Affiliations:** ^1^ Department of Terrestrial Ecology Netherlands Institute of Ecology (NIOO‐KNAW) Wageningen The Netherlands; ^2^ Laboratory of Nematology Wageningen University and Research Centre Wageningen The Netherlands

**Keywords:** litter feedback, novel communities, plant range expansion, plant–soil feedback, rhizosphere feedback

## Abstract

1. Plant–soil feedback (PSF) results from the net legacy effect that plants leave in the composition of soil communities and abiotic soil properties. PSF is induced by the rhizosphere and by litter inputs into the soil, however, we have little understanding of their individual contributions. Here, we examine feedback effects from the rhizosphere of living plants, decomposing litter and their combination.
2. We used four pairs of climate warming‐induced range‐expanding plant species and congeneric natives, and examined PSF effects on plant biomass production, as well as on decomposition in their new range.
3. We tested the hypothesis that the plant rhizosphere provides less negative feedback to range‐expanders than to the congeneric natives, and that feedback mediated by litter decomposition does not provide such a difference because decomposers might be less specialized than pathogens. To determine PSF, we used soil from the congener species within each pair as an ‘away’ soil to indicate whether range‐expanders may have lost their specialized soil biota upon arrival in the novel range.
4. Our results show that although range‐expanding plant species and their congeneric natives developed neutral PSF in both rhizosphere‐ and litter‐conditioned soils, two of the four range‐expanders produced more biomass than natives in soils conditioned by litter, that is, soils with high nutrient content. Shoot litter from two out of four range‐expanding species decomposed more than that of natives, but decomposition was unaffected by soil conditioning.
5. *Synthesis*. We compared PSF effects of range‐expanders and congeneric natives mediated via both the rhizosphere and litter using the congeneric species as a control. Under those conditions, PSF effects were neutral and not affected by plant origin. Therefore, we conclude that studies not comparing within plant genera may overestimate the impact of plant origin on PSF. Still, even under those conditions range‐expanders appeared to benefit more from high soil nutrient availability than natives, thus providing a possible advantage over congeneric natives.

## Graphical abstract

We compared Plant–soil feedback (PSF) effects of range‐expanders and congeneric natives mediated via both the rhizosphere and litter using the congeneric species as a control. Under those conditions, PSF effects were neutral and not affected by plant origin. Therefore, we conclude that studies not comparing within plant genera may overestimate the impact of plant origin on PSF. Still, even under those conditions range‐expanders appeared to benefit more from high soil nutrient availability than natives, thus providing a possible advantage over congeneric natives.

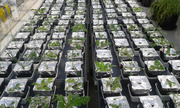

## 1 INTRODUCTION

Growing plants induce changes in the composition of soil communities and the physicochemical soil environment (Berg & Smalla, 2009). The legacy of these changes can alter the growth and fitness of plants, their neighbours and subsequent plants via so‐called plant–soil feedback (PSF) effects (Bever, Westover, & Antonovics, 1997; Kulmatiski, Beard, Stevens, & Cobbold, 2008). The biotic component of PSF is driven by three main groups of soil biota: enemies (including pathogens and herbivores), mutualistic symbionts (such as mycorrhizal fungi and rhizobia) and decomposers, which are responsible for the breakdown of organic compounds (van der Putten, Bradford, Brinkman, Voorde, & Veen, 2016). The mechanism by which these different soil biota can steer plant performance is a net effect of direct interactions with living plant roots, mostly through pathogens and mutualistic symbionts, and indirect interactions with plants via the breakdown of plant litter, mostly through decomposer organisms, or by changes in the abiotic soil environment (Wardle et al., 2004). Little is known about the contribution of these direct and indirect interactions to overall net PSF effects and subsequent plant growth responses, whereas this knowledge is relevant in order to understand how global environmental changes may influence spatio‐temporal dynamics in plant communities (van der Putten et al., 2013, 2016).

Plant–soil feedback effects are strongly influenced by the rhizosphere, which harbours a large diversity of soil micro‐organisms and invertebrates that can have direct beneficial, neutral or harmful effects on plants (Philippot, Raaijmakers, Lemanceau, & Putten, 2013; Raaijmakers, Paulitz, Steinberg, Alabouvette, & Moënne‐Loccoz, 2009; Singh, Millard, Whiteley, & Murrell, 2004). Organisms in the rhizosphere use living plant roots or root exudates released by plants, both serving as carbon and nutrient sources and, in turn, affect plant performance by feeding damage but also by regulating water and nutrient availability and the production of volatile organic compounds (Bailly & Weisskopf, 2012; Mendes, Garbeva, & Raaijmakers, 2013). In this way plants leave behind a legacy effect in the soil that may affect subsequent plant growth. Many previous studies have focused on rhizosphere‐induced PSF effects and showed that these can range from negative (pathogens and parasites dominate) to positive (mutualistic symbionts dominate) (Kulmatiski et al., 2008; Lekberg et al., 2018; Ma, Pineda, Wurff, Raaijmakers, & Bezemer, 2017).

Plant–soil feedback effects can as well be affected by plant litter decomposition (Ehrenfeld, Ravit, & Elgersma, 2005; Eppinga, Kaproth, Collins, & Molofsky, 2011; Eppinga & Molofsky, 2013; Zhang, Putten, & Veen, 2016). Besides shaping a specific rhizosphere community of soil biota, plant species may also develop distinct and specific decomposer communities (Elgersma, Yu, Vor, & Ehrenfeld, 2012; Keiser, Strickland, Fierer, & Bradford, 2011) via the quantity, quality (i.e. chemical properties) and timing of litter inputs into the soil (Austin, Vivanco, González‐Arzac, & Pérez, 2014). As a result, decomposing plant litter may leave a plant species‐specific legacy in the form of altered nutrient status, secondary chemistry, water availability and altered physical soil properties (Ehrenfeld, 2010; Freschet et al., 2013). This legacy indirectly determines subsequent plant performance of both conspecific and heterospecific plants and, therefore, contribute to PSF effects. Additionally, litter‐derived PSF may also be caused by facultative pathogens or symbiotic mutualistic organisms. Even though litter‐mediated effects on soil community composition, functioning and the resulting PSF effects are expected to be less specific than those mediated via the rhizosphere (Ke, Miki, & Ding, 2015), we still have poor understanding of the impact of plant litter legacies and their contribution to net PSF effects. For example, the decomposer pathway may reduce negative effects mediated via the rhizosphere pathway (Zhang et al., 2016). Also, upon being released from pathogens in the new range, introduced species from high‐resource availability habitats that undergo strong pathogen regulation in the original range may benefit over natives from nutrient‐rich environments in the invaded range (Blumenthal, 2006; Blumenthal, Mitchell, Pyšek, & Jarošík, 2009).Therefore, in order to further understand how PSF impacts plant growth, it will be important to take both effects mediated by rhizosphere and litter decomposition into account.

PSF effects have been proposed to play an important role during the establishment of introduced exotic plant species (Blumenthal et al., 2009; Eppinga et al., 2011; Klironomos, 2002). PSF is often less negative or more positive in novel than in native ranges (Callaway, Thelen, Rodriguez, & Holben, 2004; Reinhart, Packer, Putten, & Clay, 2003) and soil biota in the new range may alter plant resource allocation strategies (te Beest, Stevens, Olff, & Putten, 2009). Thus far, most PSF studies on introduced plants have considered exotic species that have been introduced from other continents (Suding et al., 2013). However, many plant species are also migrating intra‐continentally, because ongoing climate warming enables range expansion to higher latitudes and altitudes (Alexander, Diez, & Levine, 2015; Chen, Hill, Ohlemuller, Roy, & Thomas, 2011; Parmesan & Yohe, 2003). During these intra‐continental range shifts, PSF effects may vary between the new and original range, because of different dispersal rates of plants and soil biota (Berg et al., 2010) and different specificities of soil biota involved in rhizosphere and litter‐induced feedback interactions (van der Putten, 2012).

Range‐expanding plant species have been shown to benefit from escaping their natural enemies in the rhizosphere, thereby experiencing less negative, or even positive PSF in the new compared to the original range (Dostálek, Münzbergová, Kladivová, & Macel, 2016; Van Grunsven, Putten, Martijn Bezemer, Berendse, & Veenendaal, 2010). In addition, range‐expanders may increase soil nitrogen availability (Meisner, Boer, Cornelissen, & Putten, 2012) and may have different nutrient acquisition strategies or time of peak growth than natives (Mariotte, Spotswood, Farrer, Suding, & Fraser, 2017). Altogether, plants may respond to a specific litter legacy in the soil via nutrient availability, as well as via rhizosphere and decomposer soil communities. In order to further understand how direct effects from pathogens and mutualistic symbionts and indirect effects from decomposer organisms may contribute to PSF, a first step is to consider feedback effects from rhizosphere and litter both separately and in combination. We will do this by comparing range‐expanders in their new range with co‐occurring congeneric native species, as these range‐expanders and natives are expected to differ in the degree to which direct and indirect PSF components contribute to net PSF effects (van der Putten, 2012).

We designed a two‐phase feedback experiment in the greenhouse in order to determine PSF effects of range‐expanding plant species and congeneric‐related species that are native in the new range. Eight plant species (four range‐expanders and four natives) were grown in soils conditioned by living plants and/or plant litter. We tested four hypotheses: (1) In the conditioning phase, plant biomass will be higher in soils where litter was added than in soils without, particularly for range‐expanding plant species because of litter providing additional nutrients (Mariotte et al., 2017; Meisner et al., 2012). (2) For rhizosphere feedback effects, range‐expanders will not show different growth responses to soils conditioned by either conspecifics or congeneric natives, whereas natives will perform worst in their own conditioned soil. (3) For litter feedback effects, both range‐expanders and congeneric natives will perform best in soils conditioned by litter of range‐expanders, as range‐expanders are expected to have higher quality litter and decompose more than litter of natives (Meisner et al., 2012). (4) In the feedback phase, litter of native plant species will decompose most in their own conditioned soils, whereas no such effect is expected to occur for range‐expanders, due to the lack of specialized decomposers in the new range.

In order to test the hypotheses, we have assessed the functional capacity of the soil community, soil nutrient status, plant growth and decomposition processes in soils conditioned by living plants and/or litter. We used four pairs of range‐expanding and congeneric‐related native plant species. In our experiment, the control for PSF effects was based on comparing performance in conspecific‐conditioned soil to that in congeneric‐conditioned soil in order to detect eventual intra‐genus specialization of the soil communities between natives and range‐expanders. This method tests PSF effects of plant origin within genera, which is rare, if ever applied in studies on introduced invasive plant species. The drawback of this approach is that we are not able to detect whether range‐expanders in general have less negative PSF than congeneric natives, as done in other studies on plant range shifts (Dostálek et al., 2016; Engelkes et al., 2008; Van Grunsven et al., 2010). Nevertheless, this approach allowed us to consider PSF effects of only living plant roots, only decomposing litter, or both.

## 2 MATERIALS AND METHODS

### 2.1 Plant species selection

We used four pairs of range‐expanding plant species and congeneric natives (*Centaurea stoebe* and *Centaurea jacea*, *Geranium pyrenaicum* and *Geranium molle*, *Tragopogon dubius* and *Tragopogon pratensis*, *Rorippa austriaca* and *Rorippa sylvestris* respectively). We selected range‐expanding plant species that are present in the Netherlands and co‐occur in the same ecosystem with an abundant native plant species of the same genus (named congener). The range‐expanding plant species were first recorded in the Netherlands during the second half of the 20th century (with the exception of *Geranium*, which was first recorded in 19th century) and show an increasing trend in abundance in the Netherlands over the last decades (NDFF, 2018). Seeds were collected from field populations or purchased from a commercial supplier (Cruydt Hoeck, Nijeberkoop, the Netherlands) that harvests seeds from plant material originally collected from the field. All seeds were surface sterilized (3 min, 10% bleach solution) and germinated on sterilized glass beads under controlled conditions (20/10°C day/night, 16 hr photoperiod). Due to their small size, *Rorippa* seeds were germinated directly in gamma‐sterilized soil under the same controlled conditions (minimally 25 KGray, Syngenta BV, Ede, the Netherlands).

### 2.2 Soil collection

Background soil was collected from a former agricultural field in a riparian area near Beneden‐Leeuwen, the Netherlands (N51° 53.952, E05° 33.670) and sterilized by gamma irradiation (minimally 25 KGray, Syngenta BV, Ede, the Netherlands). Inocula soils were collected from natural field populations of each of the plant species used. We collected soil inocula from five independent populations of each plant species obtaining a total of 5 replicates × 2 plant origins (i.e., native and range‐expander) × 4 plant species pairs = 40 independent soil inocula (Table S1). In order to obtain a representative soil community for our greenhouse experiment, we created soil inocula mixes containing soil from all plant species. Soil inocula of each of the five independent replicates consisted of equal amounts of soil from each of the eight plant species (dry w/w). So, the soil inoculum for replicate one contained equal amounts of soil of replicate one of all eight plant species, the same for replicate two, three, four and five. In this way, we created five independent soil replicates, each consisting of a mix of field soil communities from all eight plant species.

### 2.3 Experimental design

The experiment consisted of two phases: a conditioning phase and a feedback phase. In the conditioning phase, we used three different types of soil conditioning treatments: with living plants (plant conditioning), with plant litter material (litter conditioning) and with both (plant + litter conditioning). This resulted into 120 pots (8 plant species × 3 soil‐conditioning types of plant, litter, and both × 5 replicates). After 13 weeks of soil conditioning, we harvested the plant material and analysed both soil nutrients and the respiration response of the soil community to added carbon substrates. The remaining soil was used to grow plants in the feedback phase.

In the feedback phase, we conducted two separate, simultaneously running experiments, one focusing on plant growth responses to soil conditioning and one focussing on litter decomposition responses to soil conditioning. For the feedback experiment on plant growth, we grew individual plants of each plant species in ‘home’ soils (i.e. conditioned by themselves) and in ‘away’ soils (i.e. conditioned by their congeneric species). There were 240 pots to test plant biomass production (3 soil‐conditioning types × 2 home/away soils × 8 plant species × 5 replicates). For the feedback experiment on litter decomposition, we used soils conditioned by plant litter material (litter conditioning) to determine the effect of species‐specific conditioning on breakdown of shoot and root litter of the same plant species and the congeneric species. The feedback phase included 200 incubation microcosms (2 shoot/root litter × 2 home/away soil × 8 plant species × 5 replicates + 40 control soil only). Control soils were incubations containing only the 40 conditioned soils (8 plant species × 5 replicates) and with no litter added, which allowed us to quantify and correct for the basal respiration of the soil.

### 2.4 Conditioning phase

We filled 3 L pots with sterilized soil mixed with 10% of live soil inocula (dry w/w). All soils were kept at 60% of the water holding capacity by watering to predetermined weight twice a week. We incubated the pots with the soil in the greenhouse for 11 days to allow the soil inocula to establish before starting the soil conditioning by plants, litter and plants + litter. We used litter of both shoots and roots, which was harvested from senescing greenhouse‐grown plants. Prior to the start of the experiment, we grew all eight plant species in sterilized soil under greenhouse‐controlled conditions (21/16°C, 16 hr photoperiod) (Table S2). When plants senesced, we harvested their shoot and root litter to use in the soil‐conditioning phase of this experiment. Root material was washed and both shoots and roots were air‐dried and sterilized prior to use in the experiment (minimally 25 KGray, Syngenta BV, Ede, the Netherlands). We fragmented litter in pieces of approx. 1 × 1 cm^2^ and added in total 6 g of dry litter per pot (50%–50% shoot–root litter). For root litter, we cut the coarse roots into segments of 1 cm when possible, and we cut fragments of approx. 1 × 1 cm^2^ when air‐dried roots formed a network of fine and tangled roots. The amount of litter was chosen to be representative of the average litter fall to the soil in temperate ecosystems (Peñuelas et al., 2007). Litter was mixed through the soil for each individual pot separately. Soils in the pots that received no litter were mixed in the same mechanical way to make all soils exposed to the same degree of disturbance. Subsequently, we planted three individuals of each plant species per pot and grew them for 13 weeks under controlled greenhouse conditions (21/16°C, 16 hr photoperiod supplied with 600 W high pressure sodium lamps at 250 µmol m^−2^ s^−1^ PAR and average air humidity of 60%).

After 13 weeks of soil conditioning, we removed the plants from the soil, washed the roots, dried both above‐ and below‐ground biomass at 40°C for 5 days and measured biomass. We immediately took a subsample of soil from each pot to analyse the soil catabolic response profile at the day of harvest and followed the protocol of Fierer et al. (2012). We added eight different organic substrates of varying complexity – labile substrates were glucose, sucrose, glycine, oxalic acid, citric acid and yeast, and recalcitrant substrates were chitin and cellulose – separately to 4 g of soil (dry weight equivalent). Soils were weighed into 50 ml centrifuge tubes modified with a rubber O‐ring in the inner part of the lid to ensure air tightness, and equipped the lids with a butyl rubber to allow headspace air sampling with a syringe. We then added 8 ml of substrate solution with concentrations ranging from 15 to 100 mM depending on the compound as used by Fierer et al. (2012). We capped the vials tightly, flushed the headspace with O_2_‐free air during 2 min at 1 bar (Westfalen Gassen Nederland BV) and incubated the vials at 20°C and darkness using a controlled climate chamber (Economic Lux chamber, Snijders Labs). We measured the net accumulation of CO_2_ over a period of 4 hr for labile substrates or 24 hr for recalcitrant substrates. A headspace sample of 6.2 ml was collected from each tube using a syringe and stored it into pre‐evacuated 5.9 ml Exetainer vial (Labco Ltd).

The concentration of CO_2_ in the gas vials (over pressure of 1 bar) was measured by injecting 250 µl of each sample in a Trace Ultra GC gas chromatograph equipped with a flame ionization detector with methanizer (mFID) (Interscience BV) and a TriplusRSH auto‐sampler (Interscience BV), and a Rt‐QBOND (30 m, 0.32 mm ID) capillary column (Restek). We used helium 5.0 as a carrier gas, a sample split ratio of 1:20 and set oven temperature at 50°C with a flow of 5 ml per minute. We used a calibration curve of known concentrations of CO_2_ ranging from 0 to 4,600 ppm of CO_2_ prepared out of a reference gas (2.38% CO_2_ in synthetic air, Westfalen AG) to determine the amount of CO_2_ in our samples. Chromeleon 7.2 Data System Software (Thermo Scientific Waltham) was used to automatize the measurements and process data.

A soil subsample was dried at 40°C for 5 days to determine moisture content and phosphorous availability (P‐Olsen) using extraction in a 0.5 M NaHCO_3_ solution and quantification by autoanalyzer (QuAAtro Autoanalyzer, SEAL Analytical Ltd). Lastly, we took a fresh soil subsample and measured mineral nitrogen and pH after KCl extraction. Briefly, we mixed 10 g of soil (based on dry weight equivalent) with 50 ml of 1 M KCl solution in a glass vial and shook it for 2 hr. We measured the concentration of mineral nitrogen (${\text{NH}}_{4}^{+}$ and ${\text{NO}}_{3}^{-}-{\text{NO}}_{2}^{-}$) in the solution using an autoanalyzer (QuAAtro Autoanalyzer, SEAL Analytical Ltd.). The remaining soil from each pot was kept in a separate bag at 4°C to be used for the two feedback phases.

### 2.5 Feedback phase: Effects on plant biomass

The feedback phase to test plant growth responses to the conditioned soils was set up 10 days after the end of the soil‐conditioning phase. We divided the soil from each pot in the soil‐conditioning phase into two parts to fill two pots of 1.1 L with the equivalent of 850 g of dry soil. We covered every pot with aluminium foil to prevent water evaporation and to diminish the number of airborne propagules that could potentially land in our conditioned soils.

We planted one seedling of each species in ‘home’ and one in ‘away’ soil. After a week, we replanted seedlings in pots where plants had failed to establish. To assess plant growth responses to the different soil‐conditioning treatments, we harvested all plants after 6 weeks of plant growth and measured shoot and root biomass after oven drying (70°C, 48 hr). We chose this relatively short duration of feedback phase to avoid both nutrient limitation and pot size limitation effects on plant growth.

### 2.6 Feedback phase: Effects on litter decomposition

After weighing the dry plant biomass resulting from the soil‐conditioning phase, we cut the shoot and root material into small pieces of 1 × 1 cm^2^ to serve as litter for a decomposition experiment. As litter originated from different plant individuals of the same species, we homogenized the resulting litter pieces within each species. Shoot and root litter were kept separately. Subsequently, we sterilized the litter material using gamma irradiation (minimally 25 KGray, Syngenta BV). After sterilization, we measured litter C:N ratio of shoots and roots of each species as a proxy for litter quality using an elemental analyser (Flash EA 1112, Thermo Fisher Scientific Inc.).

We used modified 50 ml centrifuge tubes as microcosms to measure decomposition activity. We added 1.00 g of dry sterilized litter and inoculated it with conditioned fresh soil (0.50 g of dry weight equivalent). Control microcosms were set up for each of the soils and used to quantify how much CO_2_ evolved from soil only. Due to the small amount of soil, priming effects were not measured and expected to be negligible. Microcosms were kept at 65% of the water holding capacity by watering them to predetermined weight. Water holding capacity was determined for each plant litter type and species separately.

We collected samples for CO_2_ measurements 11 times during the 48‐day incubation (on days 1, 2, 3, 5, 7, 9, 12, 17, 23, 31 and 47). We tightly closed the tubes and flushed them with CO_2_‐free air for 2 min at 1 bar. Then, tubes were placed back to the incubator for 4 hr before collecting a 6.2 ml sample of headspace air from each tube using a needle equipped with a pressure lock. We stored headspace air samples in pre‐evacuated Exetainer vials (Labco Ltd). Exetainer vials containing CO_2_ samples were stored at 4°C and darkness until their measurement in the gas chromatograph. CO_2_ concentration in the gas samples was measured using the same setting as for the catabolic response profile. However, for this case we used an additional calibration curve from 0 to 1200 ppm of CO_2_ in order to increase measurement accuracy of control samples containing only soil with no litter added. At the end of the incubation, we also assessed decomposition by measuring litter mass loss. Dry weights at the start of the incubation experiment were recorded using a 4 decimal scale, as well as on day 48 after freeze‐drying the incubation tubes with the remaining materials.

### 2.7 Data analyses

At the end of the soil‐conditioning phase, we tested whether plants used to condition the soil grew equally well with or without litter using linear mixed models with soil‐conditioning type, genera and plant origin as fixed factors and block (replicate) as a random effect factor. We assessed normality of model residuals using Q–Q plots. As all plots indicated a normal distribution of the residuals, tests were performed on untransformed data. We then used pairwise comparisons within each plant species to determine for which plant species, soil conditioning had significant effects on plant biomass. Furthermore, we analysed the effect of plant identity on soil nutrient content within each soil‐conditioning type using linear mixed effect models. Plant genera and plant origin were included as fixed factors and block (replicate) as random effect factor. We used pairwise comparisons (Tukey post‐hoc tests) within each soil‐conditioning type to test which plant genera differed from one another. Canoco 5 software was used to conduct multivariate statistics on the catabolic response profiles of the soil communities (Ter Braak, 2012). We calculated the relative mineralization of each substrate and data were log transformed prior to analyses. We used Principal Coordinate Analyses of the dissimilarity matrix based on Euclidean distances to visualize differences between soil‐conditioning treatments and plant origin within each plant pair (Crowther et al., 2014). To test the significance of soil conditioning and plant origin on the catabolic response profiles, we then performed PERMANOVA analyses (999 permutations) using the ‘adonis’ function in the ‘vegan’ package in r (Oksanen et al., 2018; R Core Team, 2017).

After the feedback phase, we analysed the effects of soil conditioning on total plant biomass production within each separate plant genus using linear mixed effect models. Soil‐conditioning type, plant origin and soil origin (home vs. away) were used as fixed effect factors and block was included as a random effect factor. Post‐hoc Tukey tests of least squared means were used to determine significant pairwise differences. We analysed litter‐derived CO_2_ production from shoot and root litter separately also using linear mixed model effects. Plant genera, plant origin and soil origin (home vs. away) were used as fixed effect factors and block was included as a random effect factor. We tested the effects of the fixed factors in all linear models with ANOVA type III testing. Linear mixed models and ANOVA tests were performed in r (R Core Team, 2017) using the package ‘lmerTest’ (Kuznetsova, Brockhoff, & Christensen, 2017). We also calculated PSF effects as ‘ln(home/away)’ (Brinkman, Putten, Bakker, & Verhoeven, 2010). We calculated PSF effects for both feedback on plant growth and on litter decomposition to assess the relative effects of soils conditioned by the congeneric species compared to soils conditioned by the conspecific in the soil‐conditioning phase. One‐sample *t* tests were used to test whether PSF effects significantly differed from 0.

## 3 RESULTS

### 3.1 Conditioning phase

#### 3.1.1 Plant biomass

**Figure 1:**
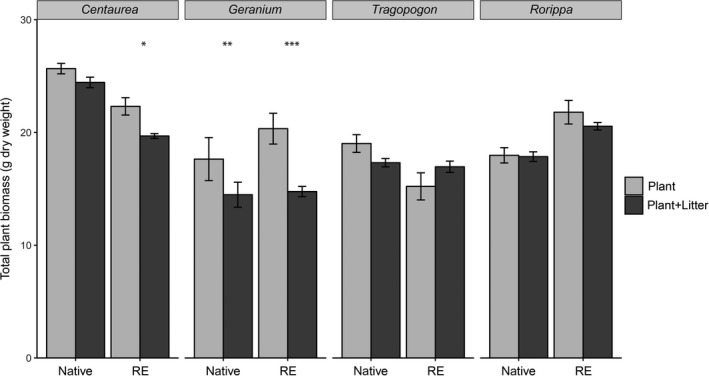
Total plant biomass of native and range‐expanding (RE) plant species at the end of the soil‐conditioning phase for the soils conditioned by living plants without litter (‘Plant’, light grey bars) and with litter (‘Plant + Litter’, dark grey bars). Error bars indicate standard error (*n* = 5). Asterisks indicate significant differences of least square means between plant biomass in the soil‐conditioning treatment with only plants and plant biomass in the soil‐conditioning treatment with plants plus litter (significance levels: ***p < .001; **p < .01; *p < .05)

Plant biomass at the end of the soil‐conditioning phase was significantly different between plant species (*F*_1,60_ = 34.1, *p* < .000) and significantly affected by the presence of plant litter in the soil (*F*_1,60_ = 21.8, *p* < .000). Furthermore, there was a significant interaction of plant species and litter presence (*F*_7,60_ = 4.22, *p* < .000). For most plant species, plant biomass during the soil‐conditioning phase did not differ between treatments with or without litter. However, plant biomass of both *Geranium* species and the range‐expander *Centaurea* were significantly greater when plants grew without litter in the soil (Figure 1).

At the end of the soil‐conditioning phase, soil nutrient availability did not differ between plant origin within each conditioning treatment (Figure S1, Table S2).

#### 3.1.2 Mineral nitrogen

At the end of the soil‐conditioning phase, soil ammonium was generally low and did not differ between plant genera or plant origin within each conditioning treatment. Soil nitrate availability differed among plant genera within all conditioning treatments, but there were no significant effects of plant origin (Figure S1, Table S2).

#### 3.1.3 Plant‐available phosphorous

At the end of the soil‐conditioning phase, conditioning with the combination of living plants and litter resulted in a significant main effect of plant genus (Table S2), because soils of *Rorippa* species had significantly higher amount of plant‐available phosphorous than soils of the other plant genera (Figure S1). In the conditioning treatment with litter only, incubation of litter of native *Geranium* led to higher availability of phosphorous in the soil than litter of range‐expanding *Geranium*, whereas this was opposite for the *Rorippa* pair (Figure S1). There were no other effects of plant origin on soil phosphorous availability.

#### 3.1.4 Catabolic response profile

At the end of the soil‐conditioning phase, the catabolic response profile of the soil communities was significantly affected by the type of soil conditioning (Figure S2, Table S3). Whether soil was conditioned by plants, litter or a combination, explained 69% of the variation in catabolic response profiles (Table S3). However, neither plant genus nor plant origin significantly affected the catabolic response profile of the soil community for any of the plant pairs (Table S3).

### 3.2 Feedback effects on plant biomass

#### 3.2.1 Conditioning by plants, litter or both

**Figure 2:**
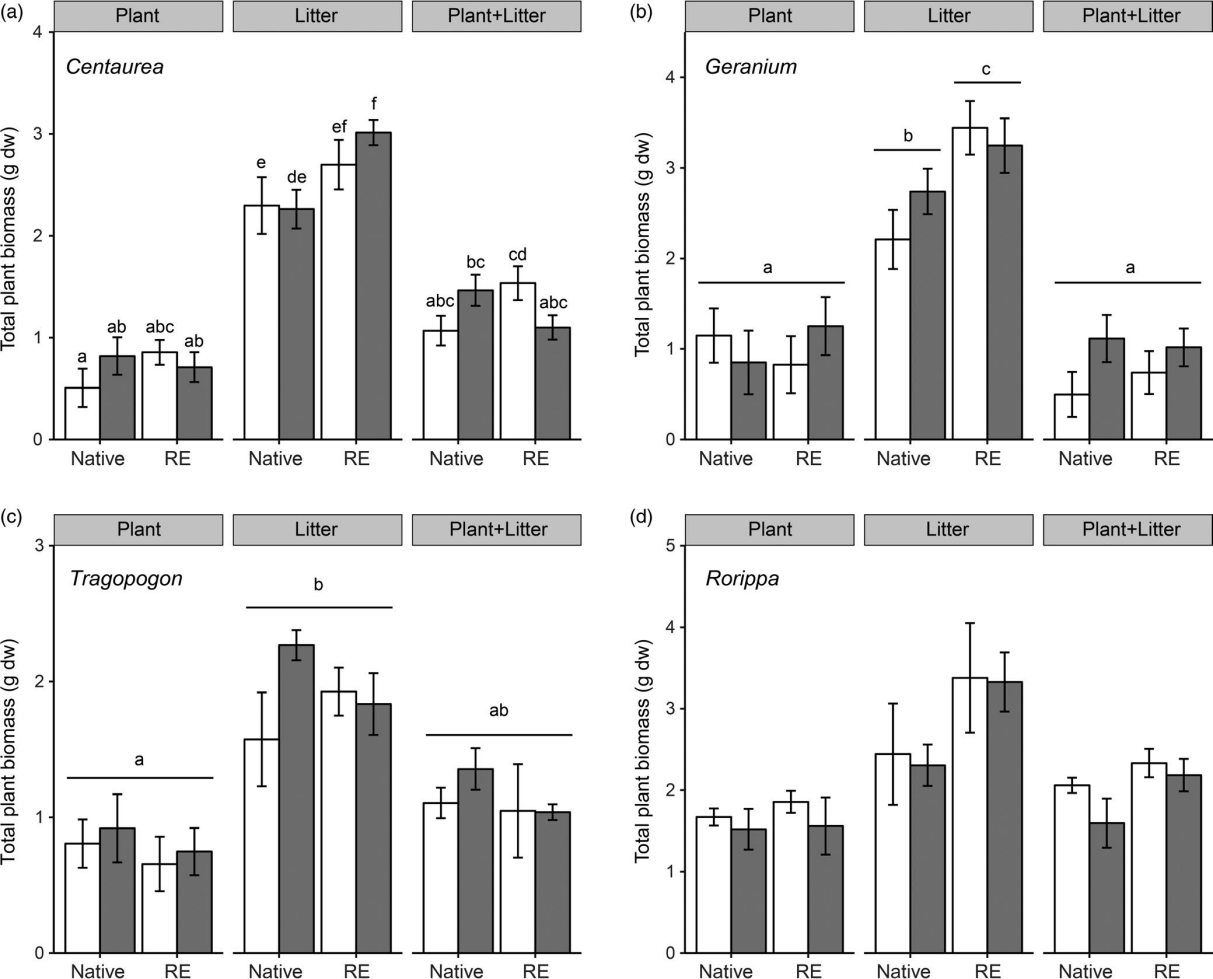
Total plant biomass at the end of the feedback phase for the native and range‐expanding (RE) plant species (a: *Centaurea*; b: *Geranium*; c: *Tragopogon*; d: *Rorippa*) grown in soils conditioned by themselves (‘home’, white bars) or soils conditioned by the congeneric species (‘away’, grey bars) and for the three types of soil conditioning (Plant, Litter, Plant + Litter). Different letters indicate significant differences (*p* < .05) between native and range‐expanding plant biomass and between soil‐conditioning treatment type (Plant, Litter, Plant + Litter). Bars are averages and error bars indicate standard error (*n* = 5)

Plants of all plant genera produced more biomass in the soils conditioned by plant litter than conditioned by living plants and by plants plus litter (Figure 2, Table 1). Range‐expanding *Centaurea* and *Geranium* produced significantly more biomass than their congeneric natives in soils that had been conditioned by plant litter (Figure 2a,b, Table 1; Soil conditioning × Plant origin interaction). In the case of *Centaurea*, there was a significant Soil conditioning × Plant origin × Soil origin interaction (Table 1, Figure 2a). This interaction appeared, because in the soil conditioned by plant litter, range‐expanders produced more biomass than natives, however, only when the range‐expander was grown in the soil of the native *Centaurea*. *Tragopogon* species produced more biomass in litter‐conditioned than in plant‐conditioned soils, while plant biomass in the combined soil‐conditioning treatment was intermediate (Figure 2c). For *Rorippa*, the main effects of soil conditioning and plant origin on plant biomass were not significant in the post‐hoc test (Figure 2d; Table 1).

**Table 1:** Linear mixed model of the effects of soil‐conditioning type (living plant rhizosphere, decomposing litter and their combination), plant origin (native and range‐expander) and soil origin (home and away) on total plant biomass at the end of the feedback phase for the four different plant genera. Fixed factors: soil‐conditioning type (C), plant origin (P) and soil origin (S)

Plant genera	Fixed factor	Total plant biomass		
		*df*	*F*	Significance
*Centaurea*	Soil conditioning (C)	2, 41	163.131	***
	Plant origin (P)	1, 41	8.912	**
	Soil origin (S)	1, 41	0.448	ns
	C × O	2, 41	4.309	*
	C × S	2, 41	0.239	ns
	O × S	1, 41	3.34	.
	C × O × S	2, 41	4.802	*
*Geranium*	Soil conditioning (C)	2, 42	92.819	***
	Plant origin (P)	1, 42	5.629	*
	Soil origin (S)	1, 42	2.695	ns
	C × O	2, 42	3.973	*
	C × S	2, 42	0.668	ns
	O × S	1, 42	0.202	ns
	C × O × S	2, 42	2.582	.
*Tragopogon*	Soil conditioning (C)	2, 38	33.74	***
	Plant origin (P)	1, 38	1.256	ns
	Soil origin (S)	1, 38	2.255	ns
	C × O	2, 38	0.172	ns
	C × S	2, 38	0.31	ns
	O × S	1, 38	1.901	ns
	C × O × S	2, 38	1.093	ns
*Rorippa*	Soil conditioning (C)	2, 41	13.072	***
	Plant origin (P)	1, 41	6.455	*
	Soil origin (S)	1, 41	1.13	ns
	C × O	2, 41	1.594	ns
	C × S	2, 41	0.073	ns
	O × S	1, 41	0.038	ns
	C × O × S	2, 41	0.107	ns

Note

Significance levels ‘***’*p* < .001; ‘**’*p* < .01; ‘*’*p* < .05.

#### 3.2.2 Soil conditioning by home versus away soils

There were no significant effects of soil conditioning by ‘home’ versus ‘away’ soils (Table 1). Only in the case of *Centaurea*, there was a significant interaction between the three factors (Soil conditioning × Plant origin × Soil origin). This interaction is mainly driven by soil conditioning by plants plus litter, which led to enhanced biomass production in the soils of range‐expanding *Centaurea* than in the soils of the native *Centaurea* (Figure 2a). In most cases, PSF effects of total plant biomass did not significantly differ from 0 in any case, indicating neutral PSF effects. There was only one exception of a negative PSF when the range‐expanding *Geranium* grew in soils conditioned by plant plus litter (Figure S3b).

### 3.3 Feedback effects on litter decomposition

**Figure 3:**
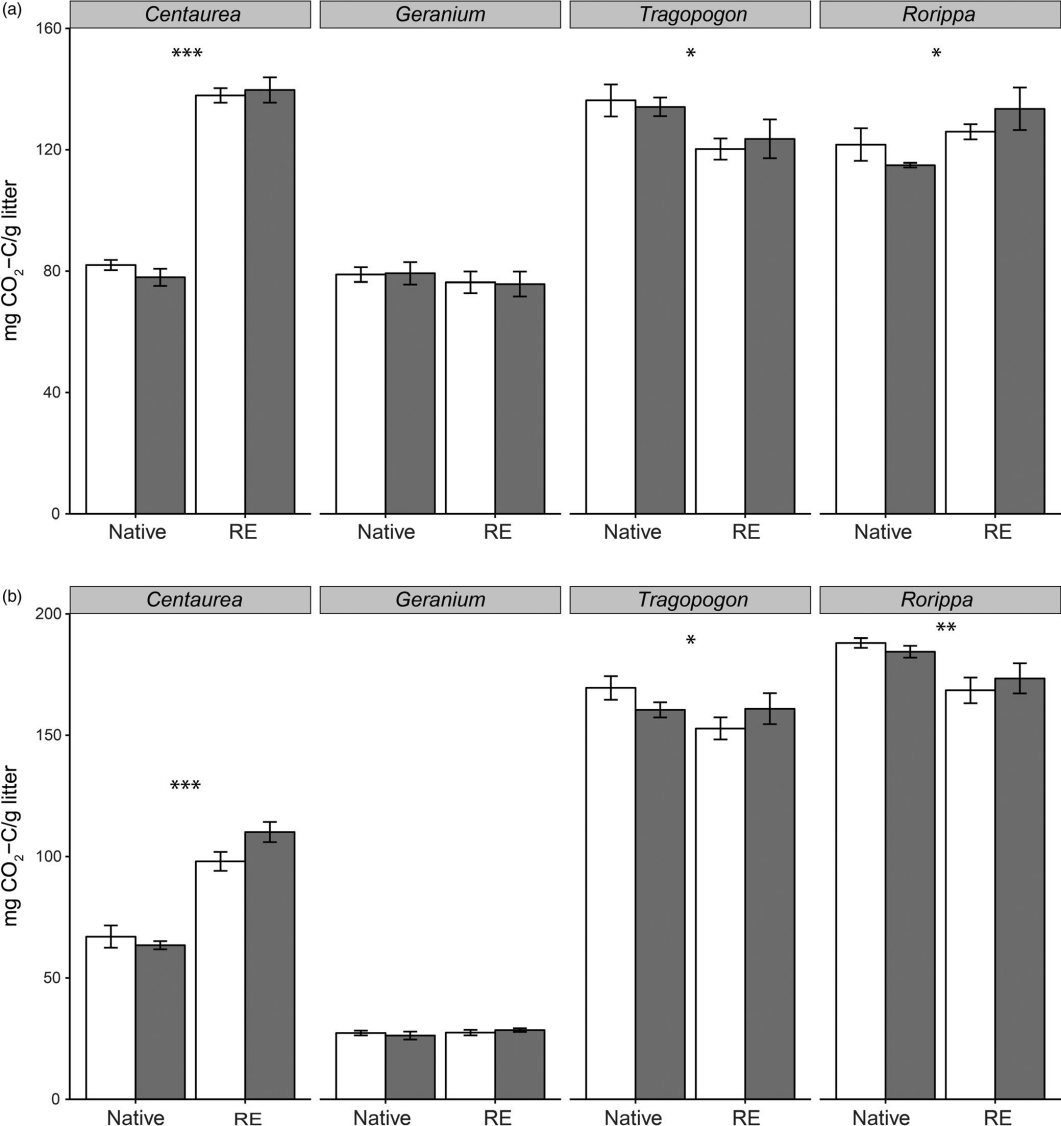
Cumulative CO_2_ mineralization from the litter incubations of shoots (a) and roots (b) of all the native and range‐expanding (RE) plant species with soils conditioned by their own species (‘home’, white bars) or by the congeneric species (‘away’, grey bars) and over a period of 48 days. Bars are averages (*n* = 5) and error bars indicate standard error. Asterisks indicate significant differences between native and range‐expanding species within plant genera (significance levels: ***p < .001; **p < .01; *p < .05)

Plant origin had a significant effect on both shoot and root litter decomposition, however, the direction of that effect varied among plant genera as indicated by the significant plant genus × plant origin interaction (Figure 3, Table 2). For instance, both shoots and roots of range‐expander *Centaurea* decomposed more than shoots and roots of its congeneric native. This effect was opposite for *Tragopogon*, where both shoots and roots of the range‐expander decomposed less than shoots of its congeneric native. In the case of *Rorippa* species, shoots of the range‐expander decomposed more than shoots of the congeneric native, however, this was the opposite for root decomposition. Decomposition of plant shoots did not depend on soil conditioning by native or range‐expander (Figure 3a, Table 2). Furthermore, roots of range‐expanders decomposed more in soils conditioned by litter of natives than of their own (Table 2; Plant origin × Soil origin interaction). Also, we show that litter breakdown measured by litter‐derived CO_2_ and by differences in weight (mass loss %, Figure S5) yielded similar results, as both variables have a strong linear relationship (Figure S6). When we calculated ‘PSF’ for litter decomposition, PSF effects were not significantly different from zero indicating that litter does not decompose differently in soils conditioned by conspecific versus congeneric species (Figure S7). Shoot litter of range‐expander *Geranium* and root litter of range‐expander *Centaurea* and *Tragopogon* had lower carbon to nitrogen ratio than their congeneric natives. This was opposite for root litter of *Geranium*, while no differences were measured for the *Rorippa* plant pair (Figure S4).

**Table 2:** ANOVA output of the linear mixed model of shoot and root decomposition at the end of the feedback phase on decomposition. Fixed factors: Genera (G), plant origin (P: native vs. range‐expander) and soil origin (S: conditioned by same species ‘home’ vs. conditioned by congeneric species ‘away’)

Fixed factors	Shoot litter			Root litter			
	*df*	*F*	Significance	*df*	*F*	Significance	
Genera (G)	3, 64	131.28	***	3, 64	1,342.97	***	
Plant origin (P)	1, 64	44.81	***	1, 64	4.73	*	
Soil origin (S)	1, 64	0	ns	1, 64	0.33	ns	
G × O	3, 64	62.77	***	3, 64	39.61	***	
G × S	3, 64	0.03	ns	3, 64	0.32	ns	
O × S	1, 64	2.35	ns	1, 64	8.04	**	
G × O × S	3, 64	0.60	ns	3, 64	0.82	ns	

Note

Significance levels ‘***’*p*< .001; ‘**’*p* < .01; ‘*’*p* < .05.

## 4 DISCUSSION

We determined feedback effects from living plant roots and decomposing litter, while analysing responses of climate warming‐induced range‐expanding and congeneric native plant species to themselves and each other. Plant–soil feedbacks are important drivers of plant community composition and dynamics (Bever et al., 1997; Kardol, Bezemer, Putten, & van der Putten, 2006) and may be caused by biotic and abiotic changes in the soil induced in the rhizosphere of living plants and by decomposing plant litter. Comparing PSFs of range‐expanding and congeneric co‐occurring native plant species to themselves and each other will help to understand and predict the establishment success and the ecological consequences of climate warming‐induced range shifts, and this knowledge may also help to understand how introduced exotic plant species will influence their new environments (Eppinga et al., 2011; van der Putten et al., 2016). Our results show that litter addition to the soil had neutral or negative effects on plant biomass depending on the plant species during the soil‐conditioning phase, and that all range‐expanding plant species and related natives developed neutral plant–soil feedbacks. Furthermore, we show that in two of the four plant pairs analysed, range‐expanders produced more biomass than natives in soils conditioned by plant litter. As litter‐conditioned soils have higher nutrient availability than soils conditioned by living plants, potentially due to plant uptake, we conclude that in half the number of cases range‐expanding plant species were able to make more use of these nutrients. In the other half, there was no difference between range‐expanders and the congeneric natives.

In contrast with our first hypothesis, the addition of plant litter to the soil during soil conditioning had no or a negative effect on conspecific plant growth (Figure 1). The biomass reducing effect of litter on *Geranium* species and range‐expander *Centaurea* in the soil‐conditioning phase could be due to facultative pathogens among the decomposer microbial community, to autotoxicity effects from conspecific litter via self‐DNA (Mazzoleni et al., 2015), or from allelopathic compounds released during decomposition (Inderjit et al., 2011). It may also be that the decomposition of *Geranium* litter caused nutrient limitation to growing plants, because of the immobilization of available nutrients by decomposers (Güsewell & Gessner, 2009). We observed that the negative effect of litter addition on plant biomass in the soil‐conditioning phase turned into a positive effect in the feedback phase, indicating that the effect of litter addition on plant growth may switch over time (Zhang et al., 2016). We did not attempt to reveal what drives the shift from negative effects of litter on plant growth during soil conditioning, and the positive effects during feedback, however, it is likely that alleviation of toxic compounds or nutrient immobilization during initial decomposition and nutrient release during ongoing decomposition may have caused this temporal litter pattern.

In support of our second hypothesis, the growth of range‐expanders in soils conditioned by living conspecifics was not different from soils conditioned by the living congenerics (Figure 2). This supports the view that, indeed, in the new range‐expanders do not have highly specialized soil biota (Van Grunsven et al., 2010). However, opposite to our second hypothesis, native plant species did not have negative PSF either (Figure S3). These neutral PSF effects of both natives and range‐expanders appear to contrast with other studies in the new range showing that range‐expanders experience less negative feedback than native species (Engelkes et al., 2008). This different result can be explained by the ‘away’ treatment that we have used in the current study, as we compared each species in soil conditioned by its own with soil conditioned by a plant species from the same genus. In other studies, the ‘away’ treatment usually consists of soil conditioned by plant species from other genera (see e.g. Engelkes et al. (2008)). We have used a so‐called congeneric‐constrained comparison as suggested by Anacker, Klironomos, Maherali, Reinhart, and Strauss (2014) and according to our results, PSF through rhizosphere legacies may not help to explain the successful establishment of range‐expanding plant species in the new range. Yet, the feedback phase in our experiment was relatively short (van de Voorde, Putten, & Bezemer, 2012). Ultimately, it will be important to test PSF of range‐expanding species and the neighbouring plant community in the field to be able to predict when and where PSF can play a role in the establishment of range‐expanding plant species.

In our third hypothesis, we expected all plants to perform best in soils conditioned by litter of range‐expanders, because litter of exotics is generally associated with higher decomposition rates and nutrient availability (Ehrenfeld et al., 2005; Eppinga et al., 2011). In contrast with our third hypothesis, our results show that plant biomass of range‐expanders and congeneric natives was not significantly different in soils conditioned by litter from either range‐expanders or natives (Figure 2, Table 1). As a result, PSF effects from litter of range‐expanders and congeneric natives were both neutral (Figure S3). The results are in line with the availability of nitrogen in the soil, which was overall high but not significantly different between soils that received range‐expander or native litters. In a previous study on plant range shifts it was shown that soils with litter from exotics had enriched available nutrients compared to related natives (Meisner et al., 2012), however, some of those range‐expanders were from intercontinental origin. The only overlapping plant genus, *Rorippa,* showed identical results in both studies.

In soils conditioned by plant litter, range‐expanders *Centaurea* and *Geranium* produced more biomass than their congeneric natives (Figure 2). This indicates that some range‐expanders, as exotic species, may be more efficient in nutrient utilization than natives (Liu & van Kleunen, 2017), which might provide them with a competitive advantage over natives. However, to conclude if that is actually the case, both range‐expanders and natives need to be grown in competition. As we did not measure nutrient mineralization and plant uptake, we cannot conclude about the role of nutrients mineralized from the litter on plant biomass responses. It has also been shown that introduced exotic species that originate from other continents can benefit from the release of soil‐borne enemies in high‐resource environments, where most plants are known to have predominant negative PSF (Blumenthal et al., 2009). Our findings suggest that this may also be the case for plants that expand their range within continents, and thereby it may be interesting to study whether synergistic effects on plant biomass may occur when including effects of both soil biota and nutrient availability.

Opposite to our fourth hypothesis, decomposer communities conditioned by litter of natives did not appear to decompose litter from natives more than from the congeneric range‐expanding species (Figure 3, Figure S7). Possibly, the plant species used do not benefit from specialized decomposers. Also, the close relationship between the natives and range‐expanders may result in decomposer communities responding to both litter types equally well (Freschet, Aerts, & Cornelissen, 2012). Thus, the plant species used in the present study may not experience as much of an advantage from specialized decomposers as plant species that have very contrasting functional traits (Veen, Freschet, Ordonez, & Wardle, 2015). While it is difficult to determine the underlying mechanisms, our analyses of the catabolic response profiles of the soil communities suggest that soils conditioned with litter of natives and range‐expanders were functionally equivalent (Figure S2, Table S3). Interestingly, the litter of range‐expander *Centaurea* (shoot and root litter) and *Rorippa* (shoot litter) had more breakdown than the litter of their native congeners (Figure 3). Taken together, these results suggest that, at least for some plant species, range‐expanders have the potential to build up a positive litter legacy effect in the soil that could promote their own performance.

## 5 SYNTHESIS

In our experiment, we found that PSF effects both through the rhizosphere of living plants and decomposing litter were neutral for both range‐expanders and congeneric‐related native plant species. The effect sizes may have been due to our approach of using ‘home’ and ‘away’ conditioned soils within plant genera. In most studies on introduced exotic plant species, PSF effects are usually not considering within‐genus comparisons. We propose that also for introduced exotic plant species it may be relevant to examine PSF effects with regards to related versus unrelated plant species that are native in the invaded range. Moreover, in our study, two out of four range‐expanding plant species benefitted disproportionally from high nutrient availability in the soil when compared to congeneric natives. Therefore, when litter of range‐expanders also decomposes at higher rates than natives, as was the case with *Centaurea* and *Rorippa* (shoots) in our experiment, range‐expanders may experience an overall positive feedback from litter decomposition when compared to native plant species from the same plant genus. We also found these results to depend on the plant species examined. In the end, however, neither range‐expanders nor natives appeared to be influenced by specialized decomposer communities. We conclude that both living plants and decomposing (above‐ground and below‐ground) plant litter contribute soil conditioning and the subsequent PSF effects. Both factors need to be considered when assessing legacies from soil conditioning and their possible contribution to the success, or failure, of intercontinental exotic plant invasions and intra‐continental range expansions.

## AUTHORS' CONTRIBUTIONS

M.M., G.F.V. and W.H.v.d.P. designed the study. M.M. and C.W. performed the experiment and collected the data. M.M. analysed the data with input from G.F.V. and W.H.v.d.P. M.M., G.F.V. and W.H.v.d.P. wrote the manuscript. All authors gave final approval for publication. Authors declare no conflict of interest. This is NIOO publication 6831.

## Data Availability

All plant biomass, soil and decomposition data have been deposited in the Dryad Digital Repository: https://doi.org/10.5061/dryad.vhhmgqnpg (Manrubia, van der Putten, Weser, & Veen, 2020).
