# Supplementary material for: Rhizosphere and litter feedbacks to range‐expanding plant species and related natives
Source: J Ecol. 2019 Oct 16;108(1):353–65. doi: 10.1111/1365-2745.13299 (PMC7363160; doi:10.1111/1365-2745.13299)
Supplement: Supplementary file 1 [file JEC-108-353-s001.docx]

**Supplementary information**

**Rhizosphere and litter feedbacks to range-expanding plant species and related natives**

M Manrubia^1^, WH van der Putten^1,2^, C Weser^1^ & GF Veen^1^

*^(1)^ Department of Terrestrial Ecology, Netherlands Institute of Ecology (NIOO-KNAW), PO Box 50, 6700 AB, Wageningen, The Netherlands; ^(2)^ Laboratory of Nematology, Wageningen University and Research Centre, PO Box 8123, 6700 ES, Wageningen, The Netherlands*

| **Table S1**. List of plant genera and species used in the experiment and the coordinates of the field locations where soils were sampled | | | | |
| --- | --- | --- | --- | --- |
| **Genera** | **Species** | **Origin** | **Site** | **Coordinates** |
| *Centaurea* | *jacea* | Native | 1 | N51° 52.076' E5° 59.529' |
|  |  |  | 2 | N51° 52.764' E6° 00.292' |
|  |  |  | 3 | N51° 52.985' E5° 42.911' |
|  |  |  | 4 | N51° 52.787' E5° 43.751' |
|  |  |  | 5 | N51° 52.036' E6° 01.505' |
|  | *stoebe ** | Range-expander | 1 | N51° 51.599' E5° 53.332' |
|  |  |  | 2 | N51° 51.605' E5° 53.332' |
|  |  |  | 3 | N51° 51.606' E5° 53.342' |
|  |  |  | 4 | N51° 51.605' E5° 53.354' |
|  |  |  | 5 | N51° 51.609' E5° 53.350' |
| *Geranium* | *molle* | Native | 1 | N51° 46.700' E5° 55.612' |
|  |  |  | 2 | N51° 52.006' E5° 59.429' |
|  |  |  | 3 | N51° 52.743' E6° 00.267' |
|  |  |  | 4 | N51° 52.627' E6° 00.032' |
|  |  |  | 5 | N51° 52.641' E6° 00.120' |
|  | *pyrenaicum* | Range-expander | 1 | N51° 57.872' E5° 40.861' |
|  |  |  | 2 | N51° 46.702' E5° 55.579' |
|  |  |  | 3 | N51° 52.662' E6° 01.296' |
|  |  |  | 4 | N51° 51.856' E6° 00.938' |
|  |  |  | 5 | N51° 51.822' E6° 00.953' |
| *Tragopogon* | *pratensis* | Native | 1 | N51° 52.972' E5° 42.850' |
|  |  |  | 2 | N51° 52.869' E5° 43.318' |
|  |  |  | 3 | N51° 52.797' E5° 43.466' |
|  |  |  | 4 | N51° 52.781' E5° 43.731' |
|  |  |  | 5 | N51° 52.468' E5° 46.793' |
|  | *dubius ** | Range-expander | 1 | N51° 50.161' E5° 51.224' |
|  |  |  | 2 | N51° 50.152' E5° 51.228' |
|  |  |  | 3 | N51° 50.285' E5° 51.113' |
|  |  |  | 4 | N51° 50.248' E5° 51.075' |
|  |  |  | 5 | N51° 50.253' E5° 51.072' |
| *Rorippa* | *sylvestris* | Native | 1 | N51° 52.275' E5° 54.398' |
|  |  |  | 2 | N51° 52.104' E5° 56.275' |
|  |  |  | 3 | N51° 52.145' E5° 59.389' |
|  |  |  | 4 | N51° 52.652' E6° 00.010' |
|  |  |  | 5 | N51° 52.761' E6° 00.255' |
|  | *austriaca* | Range-expander | 1 | N51° 52.316' E5° 54.397' |
|  |  |  | 2 | N51° 52.177' E5° 56.197' |
|  |  |  | 3 | N51° 52.057' E5° 59.462' |
|  |  |  | 4 | N51° 52.204' E5° 59.468' |
|  |  |  | 5 | N51° 52.239' E5° 59.495' |
| *(*) Range-expanders Centaurea stoebe and Tragopogon dubius are rare in the Netherlands and therefore, field soils of these species were collected from 5 individuals that were maximally 100 m apart* | | | | |

| **Table S2.** Linear mixed model analysis of the effects of plant genera and plant origin on soil pH and nutrient availability after the conditioning phase for the three types of soil conditioning: living plant rhizosphere, decomposing litter, and their combination. Fixed factors: Plant genera (G) and plant origin (O). Response variables: soil pH, soil nitrate and nitrite availability (NO_3_+NO_2_), ammonium (NH_4_) and available phosphate (PO_4_). | | | | | | | | | | |
| --- | --- | --- | --- | --- | --- | --- | --- | --- | --- | --- |
| Conditioning | Fixed factor |  | pH | | N-(NO_3_+NO_2_) | | N-(NH_4_) | | P-(PO_4_) | |
|  |  | num df | F | Signif. | F | Signif. | F | Signif. | F | Signif. |
| Plant | Genera (G) | 3 | 0.216 | ns | 10.080 | ******* | 1.181 | ns | 2.091 | ns |
|  | Origin (O) | 1 | 0.632 | ns | 1.104 | ns | 2.518 | ns | 3.715 | . |
|  | G x O | 3 | 0.039 | ns | 0.190 | ns | 0.554 | ns | 0.487 | ns |
| Litter | Genera (G) | 3 | 2.610 | . | 3.829 | ***** | 0.380 | ns | 0.620 | ns |
|  | Origin (O) | 1 | 1.618 | ns | 0.782 | ns | 1.411 | ns | 0.039 | ns |
|  | G x O | 3 | 0.740 | ns | 1.849 | ns | 1.144 | ns | 5.917 | ****** |
| Plant+Litter | Genera (G) | 3 | 2.223 | ns | 14.566 | ******* | 0.199 | ns | 7.196 | ******* |
|  | Origin (O) | 1 | 0.461 | ns | 0.563 | ns | 0.466 | ns | 0.240 | ns |
|  | G x O | 3 | 0.821 | ns | 0.391 | ns | 0.397 | ns | 1.972 | ns |
| Significance levels “***” p<0.001; “**” p<0.01; “*” p<0.05; “.” p<0.1 | | | | | | | | | | |

| **Table S3**. PERMANOVA test (9999 permutations) on Euclidean distance matrix of catabolic response profile data for all plant species and conditioning treatments: living plant rhizosphere, decomposing litter, and their combination. Fixed factors: Plant genera (G), plant origin (O) and soil conditioning (i.e. conditioned by living plants, plant litter or both, C). | | | | | | |
| --- | --- | --- | --- | --- | --- | --- |
|  | Df | SS | MS | F | R^2^ | p-value |
| Plant genera (G) | 3 | 919 | 306 | 1.288 | 0.006 | 0.274 |
| Plant origin (O) | 1 | 65 | 65 | 0.273 | 0.000 | 0.647 |
| Soil conditioning (C) | 2 | 128548 | 64274 | 270.270 | 0.839 | **0.000** |
| G x O | 3 | 184 | 61 | 0.257 | 0.001 | 0.883 |
| G x C | 6 | 285 | 47 | 0.200 | 0.002 | 0.988 |
| O x C | 2 | 89 | 44 | 0.187 | 0.001 | 0.877 |
| G x O x C | 6 | 352 | 59 | 0.247 | 0.002 | 0.973 |
| Residuals | 96 | 22830 | 238 |  | 0.149 |  |
| Total | 119 | 153271 |  |  | 1.000 |  |


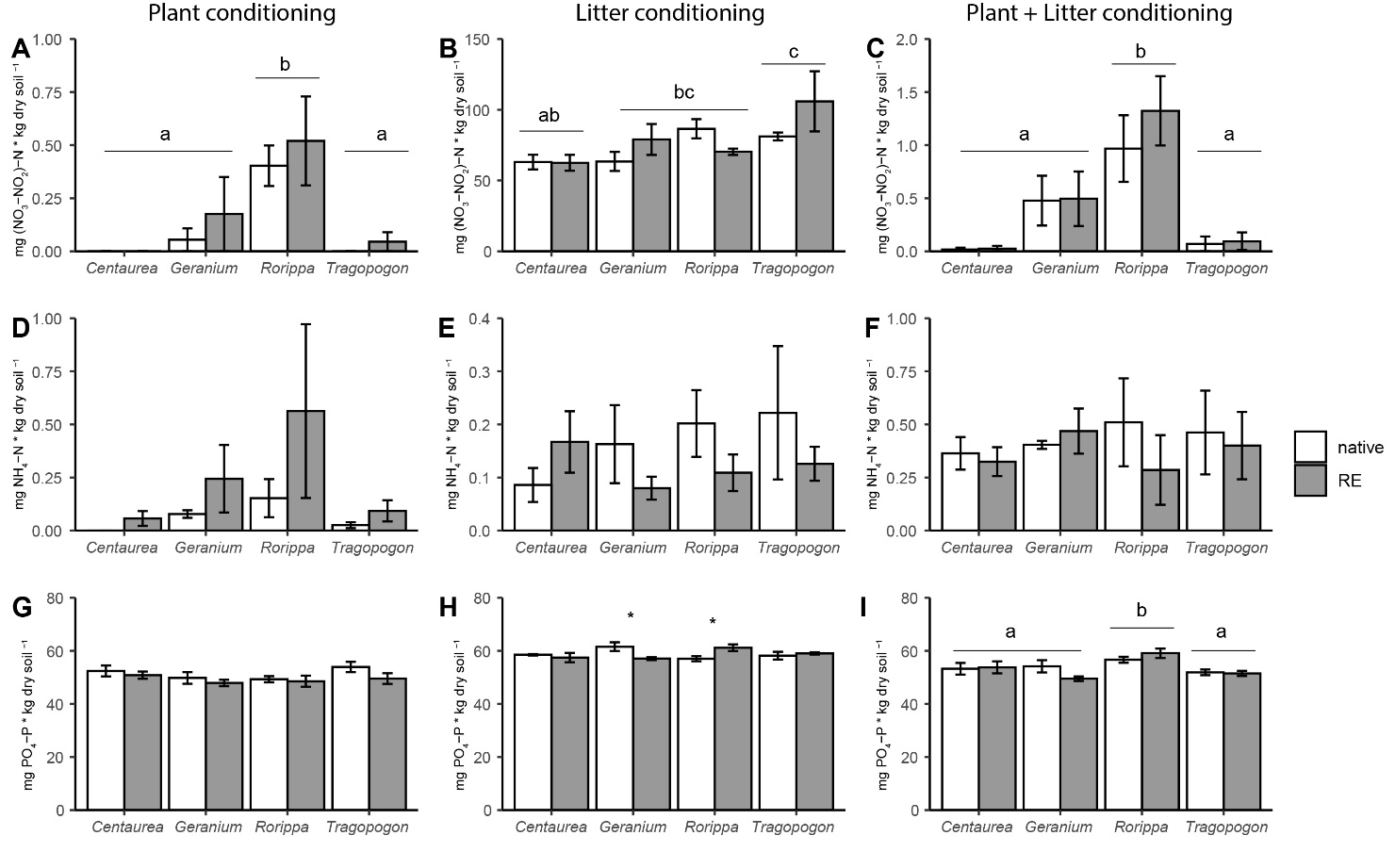


**Fig S1.** Soil nitrate and nitrite availability (A, B, C), ammonium (D, E, F) and available phosphate (G, H, I) for all plant species and soil conditioning types (Plant conditioning: A, D, G; Litter conditioning: B, E, H) and Plant + Litter conditioning (C, F, I) at the end of the conditioning phase. White bars represent native plants and grey bars represent range-expanding plants for each genera. Bars are averages per treatment ± standard error (N=5). Letters indicate significant differences between plant genera (*p*<0.05). Asterisks indicate significant differences between plant origin within plant genera (*p*<0.05). Note that y-axis have different ranges.


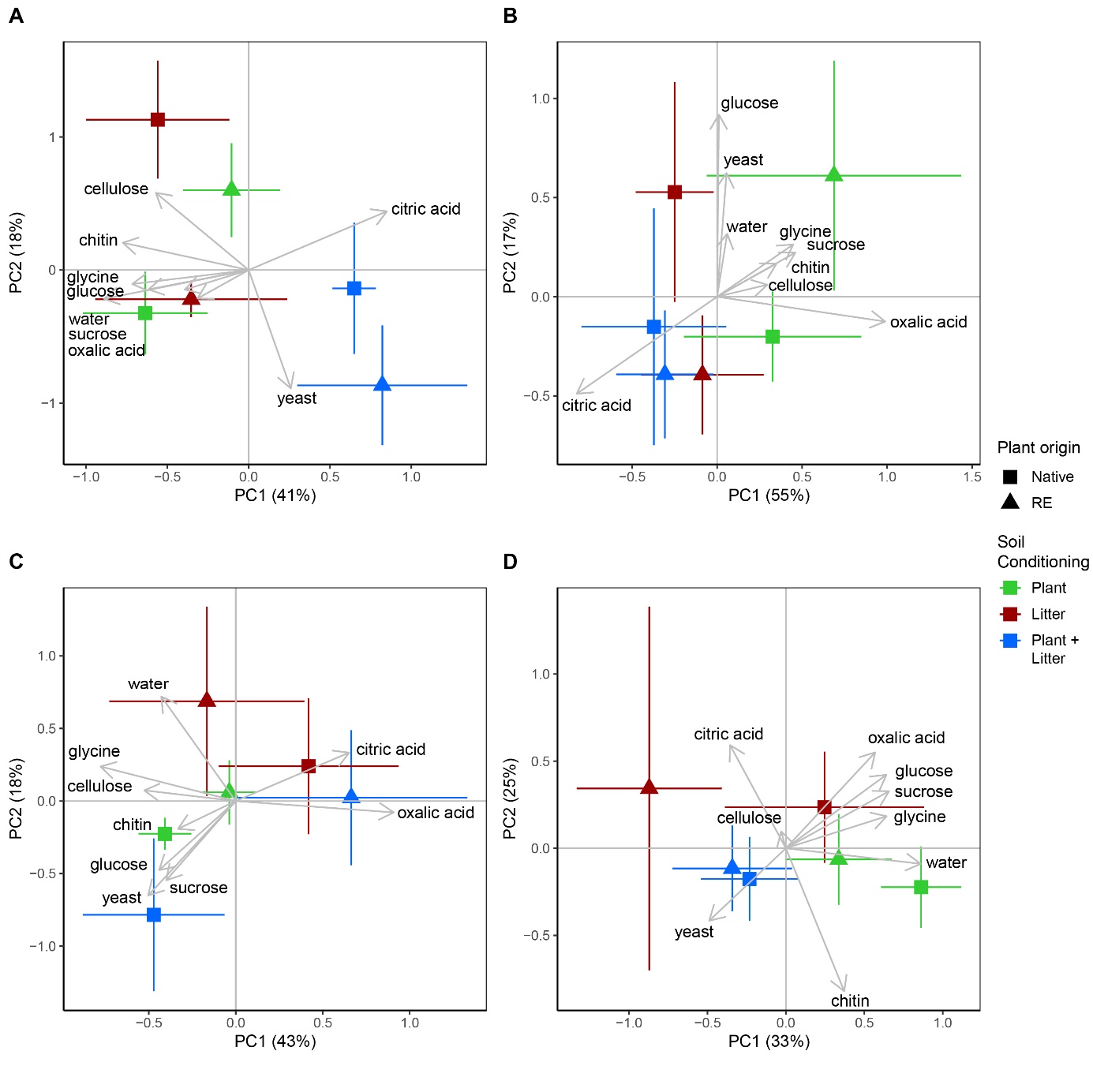


**Fig. S2**. Principal coordinate analyses of the catabolic response profiles for each plant pair (A: *Centaurea*; B: *Geranium*; C: *Tragopogon*; D: *Rorippa*). Squares represent native and triangles represent range-expanding (RE) plant species. Different colors indicate the conditioning treatments: plant (green), litter (red), plant + litter (blue). Symbols are centroids and error bars indicate standard errors (n=5).

**
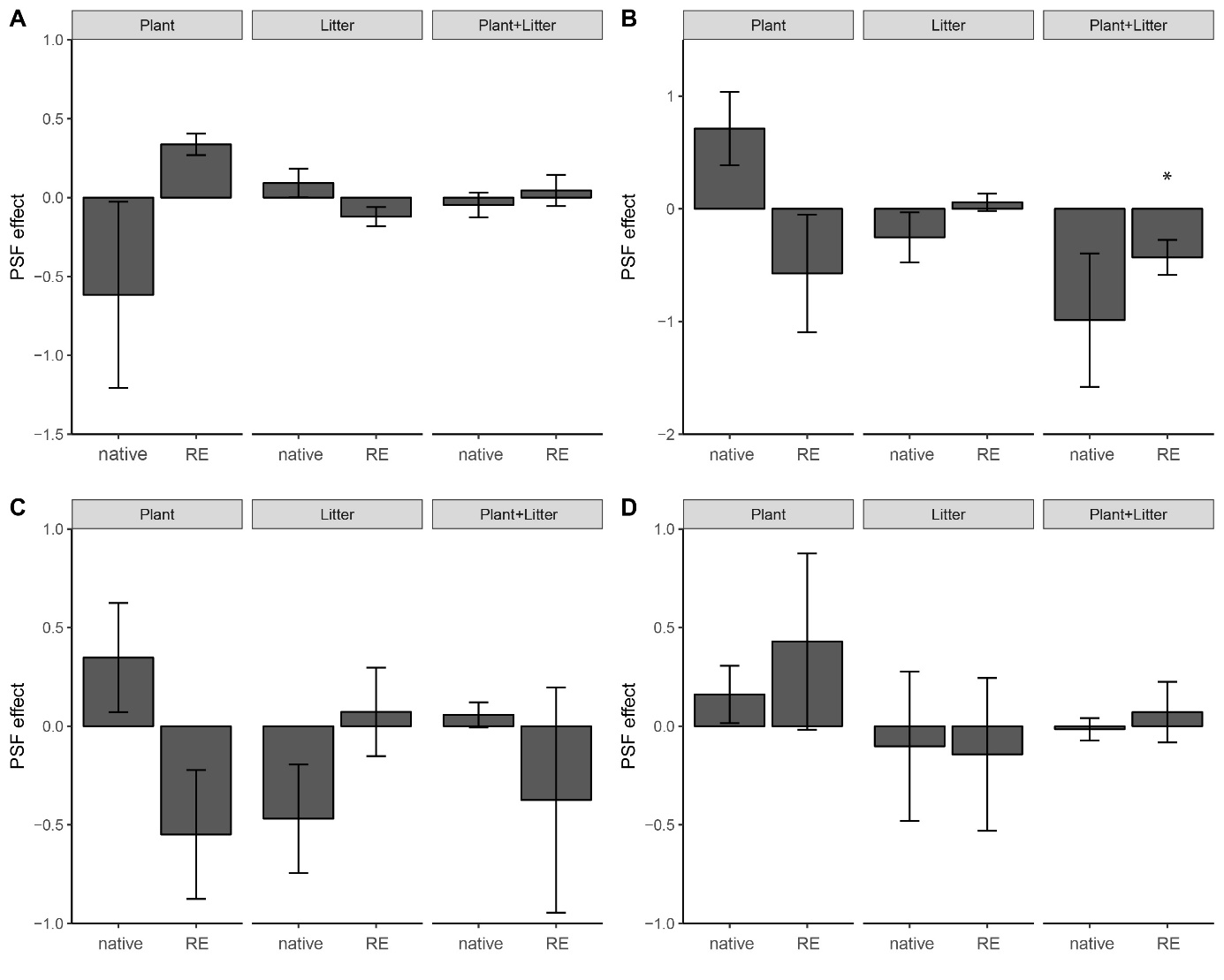
**

**Fig. S3.** Plant-soil feedback effect [ln(home/away)] on plant biomass of native and range-expanding (RE) plant species of the four genera (A: *Centaurea*; B: *Geranium*; C: *Tragopogon*; D: *Rorippa*). Bars are averages (n=5) and error bars indicate standard errors. Asterisks indicate significant difference from 0 tested with one-sample t-test. Significance levels: *** p<0.001; ** p<0.01; * p<0.05.


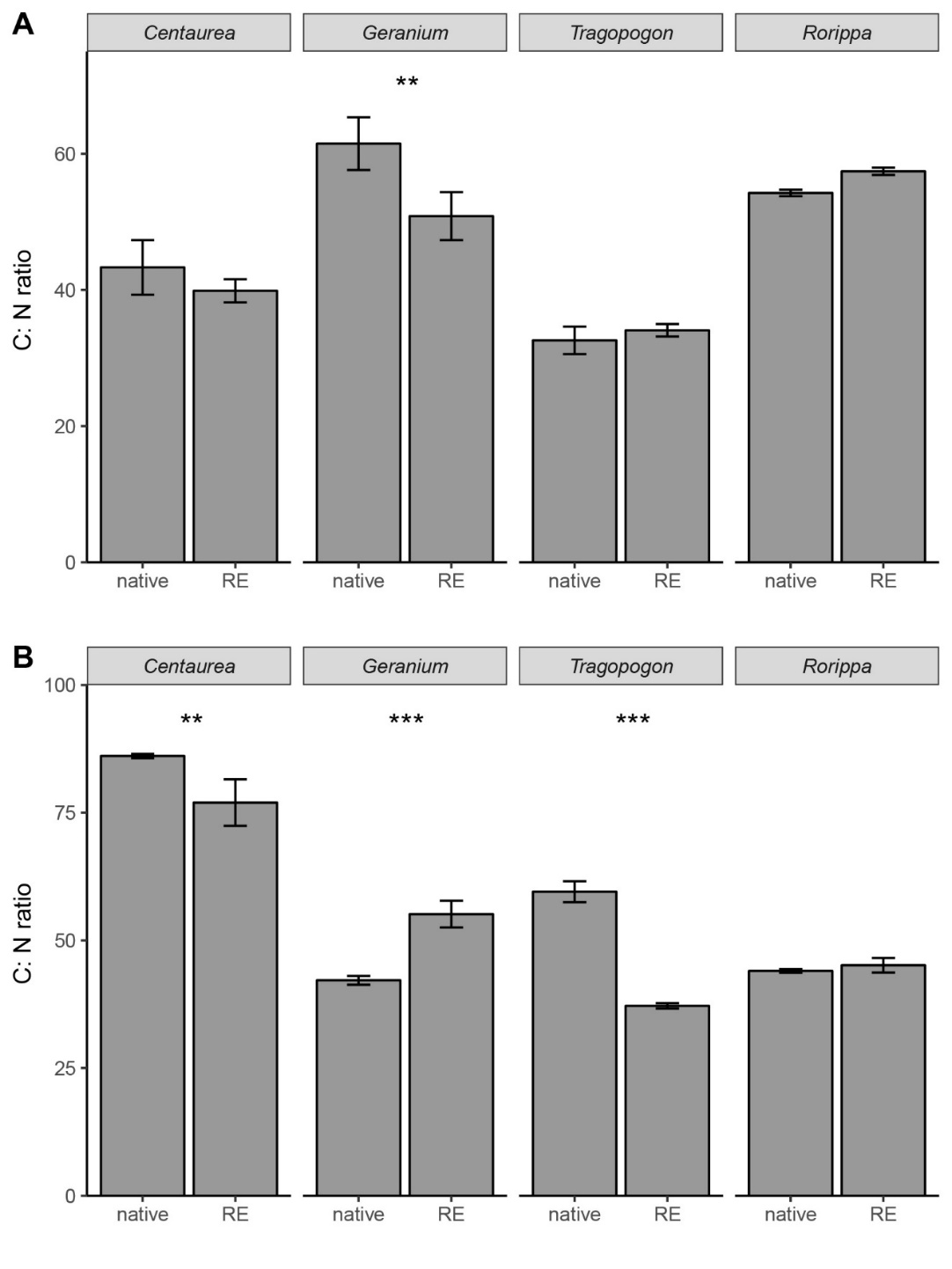


**Fig. S4.** Carbon to nitrogen ratio (C: N ratio) of shoot (A) and root (B) litter of the native and range-expander (RE) plant species used in the feedback phase to test effects on decomposition. Bars are averages (n=5) and error bars indicate standard error. Asterisks indicate significant differences in litter C: N ratio between native and range-expander within plant genera. Significance levels: *** p<0.001; ** p<0.01; * p<0.05.


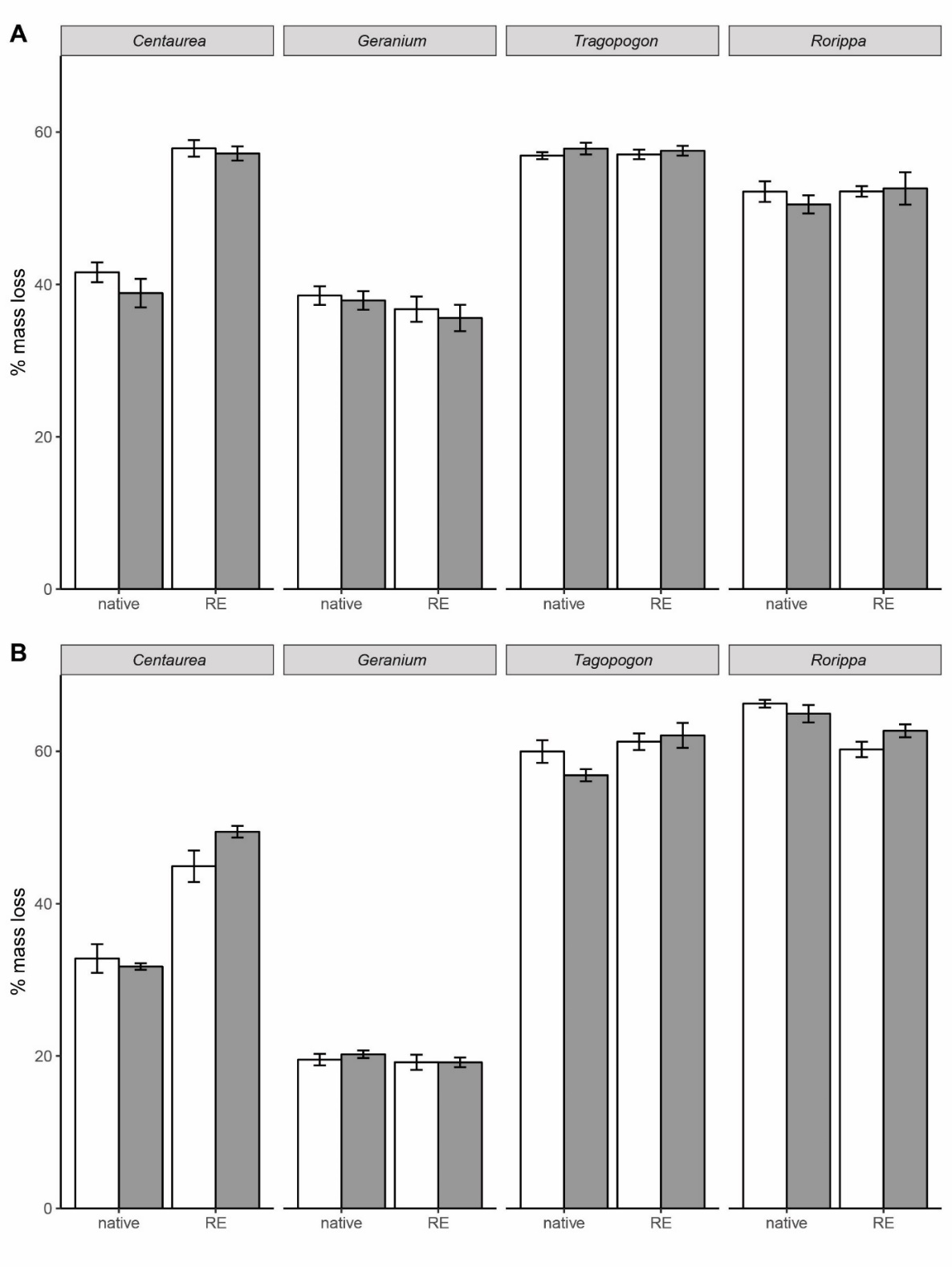


**Fig. S5.** Litter mass loss (% of mass loss) of shoots (A) and roots (B) at the end of the feedback phase to test effects on decomposition. Each litter was incubated in soils that have been conditioned by the same litter species (“home”, white bars) and in soils that have been conditioned by the congeneric plant species (“away”, grey bars). Bars are averages (n=5) and error bars indicate standard error.

**
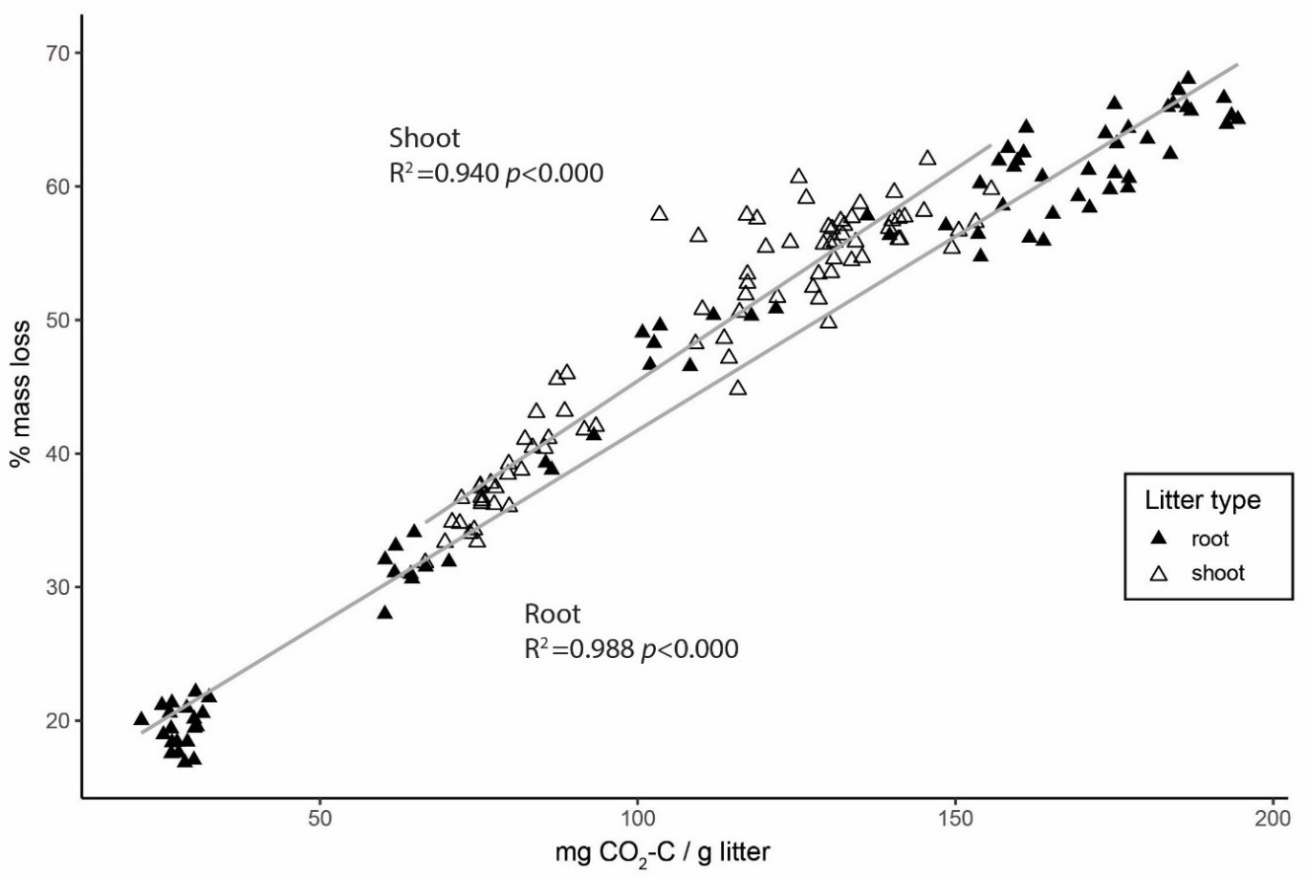
**

**Fig. S6.** Relationship between cumulative litter-derived CO_2_ and % of litter mass loss at the end of the feedback phase on decomposition for shoot (open triangles) and root litter (filled triangles). R^2^ and p-value of the Pearson correlation test are indicated for shoot and root litter separately.

**
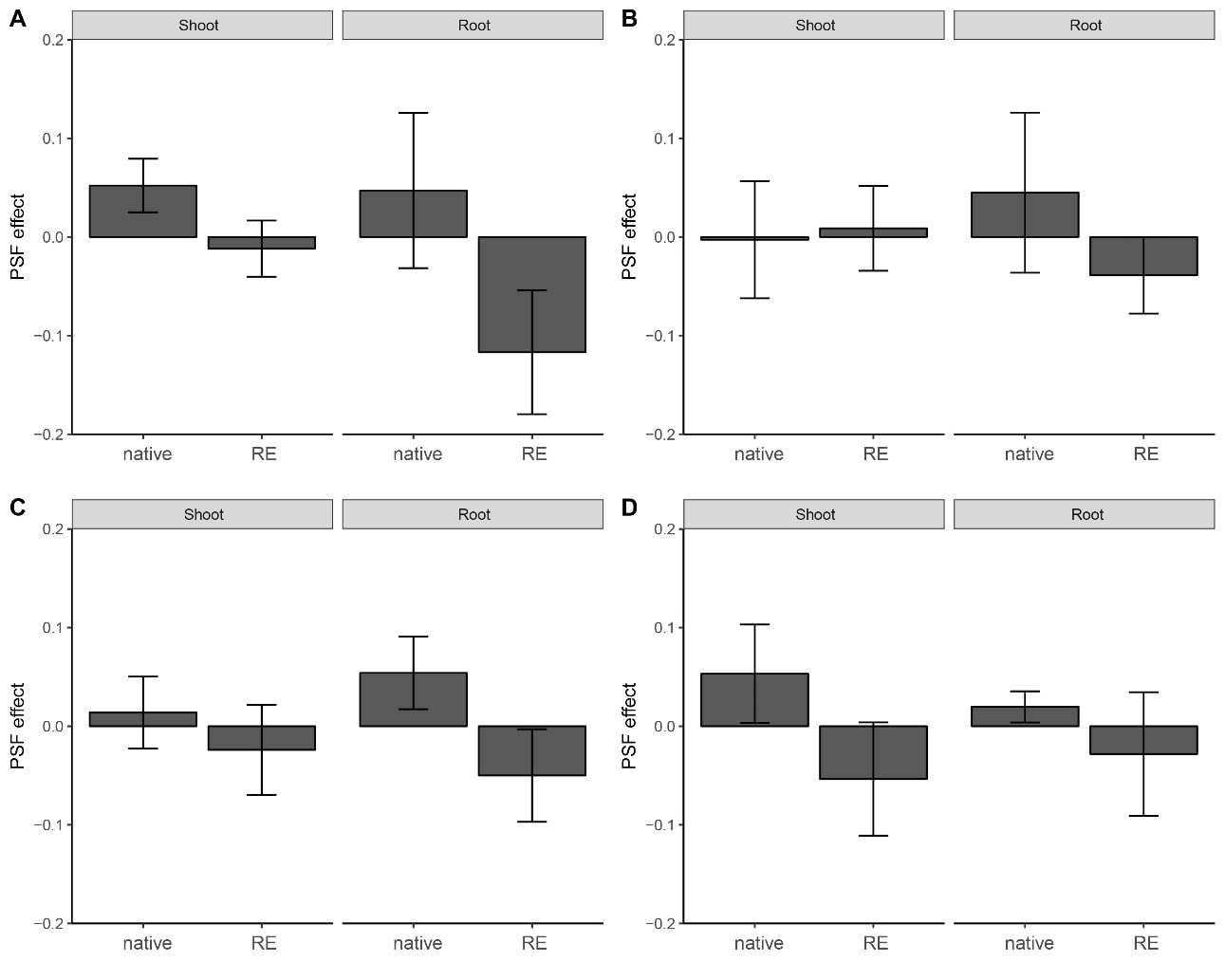
**

**Fig. S7.** Plant-soil feedback effect [ln(home/away)] on litter decomposition of native and range-expanding (RE) plant species of the four genera (A: *Centaurea*; B: *Geranium*; C: *Tragopogon*; D: *Rorippa*). Bars are averages (n=5) and error bars indicate standard errors.
